# Machine Learning-Based Prognostic Modelling Using MRI Radiomic Data in Cervical Cancer Treated with Definitive Chemoradiotherapy and Brachytherapy

**DOI:** 10.3390/curroncol32110602

**Published:** 2025-10-27

**Authors:** Kamuran Ibis, Mustafa Durmaz, Deniz Yanik, Irem Bunul, Mustafa Denizli, Erkin Akyuz, Bayarmaa Khishigsuren, Ayca Iribas Celik, Merve Gulbiz Dagoglu Kartal, Nezihe Seden Kucucuk, Inci Kizildag Yirgin, Murat Emec

**Affiliations:** 1 Department of Radiation Oncology, Institute of Oncology, Istanbul University, 34093 Istanbul, Türkiye; deniz.yanik@istanbul.edu.tr (D.Y.); mustafadenizli@istanbul.edu.tr (M.D.); erkinakyuz@istanbul.edu.tr (E.A.); ayca.iribas@istanbul.edu.tr (A.I.C.); nezihe.kucucuk@istanbul.edu.tr (N.S.K.); 2 Department of Radiology, Istanbul Faculty of Medicine, Istanbul University, 34093 Istanbul, Türkiye; mustafa.durmaz@istanbul.edu.tr (M.D.); gulbiz.kartal@istanbul.edu.tr (M.G.D.K.); 3 Department of Radiation Oncology, Erzurum City Hospital, 25240 Erzurum, Türkiye; irem.bunul@istanbul.edu.tr; 4 Department of Medical Oncology, Institute of Oncology, Istanbul University, 34093 Istanbul, Türkiye; bayarmaa.khishigsuren@istanbul.edu.tr; 5 Department of Radiology, Institute of Oncology, Istanbul University, 34093 Istanbul, Türkiye; inci.kizildagyirgin@istanbul.edu.tr; 6 Computer Science Application and Research Centre, Istanbul University, 34116 Istanbul, Türkiye; murat.emec@istanbul.edu.tr

**Keywords:** radiotherapy, uterine cervical neoplasms, machine learning, radiomics

## Abstract

**Simple Summary:**

Locally advanced cervical cancer is commonly treated with chemoradiotherapy and 3D image-guided adaptive brachytherapy (3D-IGABT). While advances in systemic therapies and radiotherapy techniques have improved survival and reduced side effects, the disease remains prevalent in low-resource settings, making accurate pretreatment prognosis increasingly important. This study evaluated CatBoost-based machine learning models for survival prediction in patients with locally advanced cervical cancer. Results showed that models integrating both clinical and radiomic features outperformed those using only clinical data, with notable improvements in accuracy and F1-score. Radiomic features, particularly from T1-weighted (T1W) and T2-weighted (T2W) MRI sequences, significantly enhanced the models’ predictive performance. The study stands out for its focus on a relatively under-researched cancer stage, its use of long-term follow-up data, and its comprehensive inclusion of patient and treatment characteristics. These findings contribute valuable insight into prognosis prediction in settings where cervical cancer is most prevalent.

**Abstract:**

Background: This study aims to evaluate the contribution of clinical and radiomic features to machine learning-based models for survival prediction in patients with locally advanced cervical cancer. Methods: Clinical and radiomic data from 161 patients were retrospectively collected from a single center. Radiomic features were obtained from contrast-enhanced magnetic resonance imaging (MRI) T1-weighted (T1W), T2-weighted (T2W), and diffusion-weighted (DWI) sequences. After data cleaning, feature engineering, and scaling, survival prediction models were created using the CatBoost algorithm with different data combinations (clinical, clinical + T1W, clinical + T2W, clinical + DWI). The performance of the models was evaluated using test accuracy, precision, recall, F1-score, ROC curve, and Bland–Altman analysis. Results: Models using both clinical and radiomic features showed significant improvements in accuracy and F1-score compared to models based solely on clinical data. In particular, the CatBoost_CLI + T2W_DMFS model achieved the best performance, with a test accuracy of 92.31% and an F1-score of 88.62 for distant metastasis-free survival prediction. ROC and Bland–Altman analyses further demonstrated that this model has high discriminative power and prediction consistency. Conclusions: The CatBoost algorithm shows high accuracy and reliability for survival prediction in locally advanced cervical cancer when clinical and radiomic features are combined. The addition of radiomics data significantly improves model performance.

## 1. Introduction

Cervical cancer is one of the most common malignancies among women worldwide and one of the leading causes of cancer-related deaths [1]. The implementation of human papillomavirus (HPV) vaccination and screening programs has shown promising reductions in the incidence of cervical cancer in many Western countries [2]. However, 85% of locally advanced cervical cancer (LACC) cases worldwide occur in low-income countries, where cervical cancer incidence is higher [3].

After studies demonstrating the benefit of chemoradiotherapy (CRT) over radiotherapy (RT), CRT followed by brachytherapy (BT) has been the standard treatment for LACC since 1999. However, a new era has begun with the results of studies investigating the addition of neoadjuvant chemotherapy (ChT), adjuvant ChT, and immunotherapy [4,5,6,7]. While various systemic therapies are being investigated, external beam radiation therapy (EBRT) and BT remain the cornerstone of treatment. Chemoradiotherapy and magnetic resonance imaging (MRI)-guided adaptive brachytherapy (IGABT) are effective in all stages of LACC and provide a limited rate of severe morbidity [8].

Magnetic resonance imaging-based target volume definition and dose optimization have provided improved clinical outcomes with reduced toxicities [9,10]. While MRI is superior for target definition, its widespread applicability, availability, and logistical and financial implications are limited. Therefore, the use of computed tomography (CT) imaging has become essential for the wider acceptance and adoption of IGABT, and IBS-GEC ESTRO-ABS guidelines for CT-based contouring in IGABT for cervical cancer have been published [11].

The higher incidence of cervical cancer, particularly in resource-limited countries, along with systemic treatment innovations, diversity, and costs, as well as challenges in accessing standard treatments enabled by technological advancements, present significant challenges. Survival prediction in LACC is of critical importance for treatment planning and follow-up of patients. Therefore, nomograms, scoring systems, and marker studies are being developed [12,13,14].

Radiomics is a rapidly evolving research area, investigating this non-invasive technique for extracting high-throughput quantitative features from medical imaging [15]. It works by analyzing a lesion’s three-dimensional texture to produce a multitude of parameters with potential connections to the tumor’s intrinsic heterogeneity, malignant potential [16]. More accurate image features can be extracted and provide clinicians with more comprehensive information to develop more precise personalized treatment procedures. Several studies have investigated the efficacy of radiomics in predicting clinical outcomes for LACC [17,18,19,20]. Wang et al. demonstrated that machine learning-driven radiomics models are effective in predicting disease-free survival and overall survival (OS) after CRT in LACC patients, with the combination of tumor and peritumor information yielding superior predictive power and supporting reliable therapeutic decision-making in cervical cancer [16].

In recent years, the combination of clinical and radiomic (image-based) features with machine learning has shown promise in improving the accuracy of survival prediction. Different machine learning algorithms (e.g., random forest, gradient boosting, support vector machines, deep learning) have demonstrated high accuracy in both classification and regression-based survival predictions [16,21,22,23,24,25,26]. Machine learning-based models facilitate the development of personalized treatment and follow-up strategies by categorizing patients into high-, medium-, and low-risk groups [16,22,23,27]. The integration of clinical and imaging data can provide significant contributions to personalized treatment planning and patient follow-up. Although a growing number of studies have evaluated the role of machine learning and radiomics in cervical cancer prognosis, consensus on optimal methodologies, reproducibility across cohorts, and integration into clinical workflows is still limited. Our study included only patients with LACC treated with CT-based 3D-IGABT following definitive CRT. It differs from other studies due to the large number of clinical and treatment features beyond the radiological images. Furthermore, although it consists of single-center data, it includes information from a geographic sample group for which less data exists in the literature.

In our study, we aimed to evaluate the contribution of clinical data (patient characteristics and treatment features) and radiomics features obtained from pretreatment MRIs in machine learning-based models in patients diagnosed with LACC who underwent simultaneous CRT followed by CT-based IGABT, and to determine the model combinations with the highest accuracy and predictive power. The novelty of this study lies in its incorporation of a long median follow-up of nearly eight years, the systematic evaluation of T1W, T2W, and DWI radiomics in combination with extensive clinical and treatment features, and its specific focus on CT-based 3D-IGABT, which provides unique insights for low-resource settings where MRI-guided brachytherapy may not be feasible.

## 2. Materials and Methods

### 2.1. Study Design and Patient Selection

A total of 161 patients with LACC who were treated with curative CRT and CT-based 3D-IGABT in our clinic between 2010 and 2021 were included in the retrospective study. The inclusion criteria for the study were patients over 18 years of age, diagnosed with LACC, not undergoing surgery, treated with definitive CRT and 3D-IGABT, having pre-treatment MRI, and follow-up. The exclusion criteria were as follows: patients who had undergone re-irradiation or radical surgery in the pelvis were not considered for inclusion. Clinical and imaging data were obtained from medical records, the hospital information management system, and archived digital images. Sociodemographic, clinical, and treatment parameters, including patient age, performance status, hemoglobin, neutrophil/lymphocyte ratio, menopausal status, histology, tumor size, 18F-FDG SUV of the primary tumor, presence of lymphadenopathy, ChT, RT, and BT characteristics (dose, fractions), MRI response of the primary tumor to CRT, presence and size of residual tissue, stage, high risk-clinical target volume (HR-CTV) at the time of the first brachytherapy application; HR-CTV D98 EQD2_10Gy_, HR-CTV D90 EQD2_10Gy_, HR-CTV D50 EQD2_10Gy_, and right and left A points EQD2_10Gy_ total doses, and recurrence status during follow-up, were recorded.

### 2.2. Treatment Characteristics

All patients underwent a pretreatment gynecological examination, and examination findings were documented. Pretreatment abdominal MRI and 18F-FDG-PET/CT imaging were used to assess local disease and lymph node involvement. Staging was performed according to the FIGO 2018 staging system using examination and imaging findings. External beam radiotherapy (3D-CRT) or IMRT/VMAT techniques were used, with a dose of 1.8–2 Gy per fraction to a total of 45–50 Gy. Concurrent chemotherapy (40 mg/m^2^ cisplatin) was administered. After CRT, an MRI was obtained to assess treatment response, and a CT-based 3D image-guided adaptive intracavitary high dose rate (HDR) brachytherapy was applied. The prescribed dose was defined as HR-CTV. The total doses obtained from HR-CTV, R, and L point A after EBRT and BT were calculated as equivalent dose in 2 Gy fractions (EQD2), assuming α/β = 10 Gy for the tumor. Treatment response was assessed with MRI and 18F-FDG-PET/CT 3 months after completion of treatment. Follow-up was performed every 3 months for the first 2 years, every 6 months up to 5 years, and annually thereafter. Patient and treatment characteristics are presented in Table 1.

### 2.3. Image Acquisition

Pre-treatment pelvic MRI scans were retrieved from our institution’s Picture Archiving and Communication System (PACS). MRI images were acquired using 1.5 Tesla MRI devices, the Philips Achieva (Philips Medical Systems, Best, The Netherlands) and Symphoni (Siemens, Erlangen, Germany) models. Axial/axial oblique (perpendicular to the long axis of the cervix) T2W, contrast-enhanced T1W, and DWI images were selected for radiomic analysis. Additionally, the contrast agent gadopentetate glucosamine injection (Gd-DTPA) was administered at a dose of 0.1–0.2 mmol/kg via the elbow vein at a rate of 1.5 mL/s. To mitigate potential interscanner variabilities arising from differences in scanner settings or acquisition parameters, subsequent data harmonization steps were applied.

### 2.4. Tumor Segmentation and Feature Extraction

**Figure 1:**
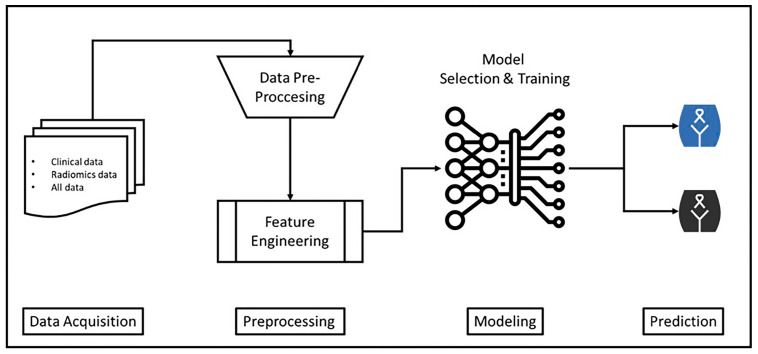
Proposed Flow Chart for Cervical Cancer Survival Prediction. Blue: No event; Black: Event.

Tumor segmentation was performed manually using commercially available LIFEx Software (version 7.8.0; www.lifexsoft.org (accessed on 3 February 2025)) [28]. The entire tumor volume (volume of interest, VOI) was segmented in post-contrast T1W, T2W, and DWI sequences. Segmentations were independently performed by two radiologists, each with 5 and 15 years of experience. In cases where lesion boundaries were confusing or inter-reader disagreement arose, a third radiologist with 20 years of experience reviewed the images. Final segmentation was established through consensus. Radiomic feature extraction was performed within the LIFEx platform following the Image Biomarker Standardization Initiative (IBSI) guidelines to ensure standardization and reproducibility [29]. To ensure reproducibility and transparency, a structured radiomics pipeline was applied. Preprocessing included intensity normalization using z-score scaling and bias field correction to minimize scanner-related intensity inhomogeneities. Low-variance features were removed, and highly correlated features (Pearson’s r > 0.9) were excluded to reduce redundancy. Subsequently, feature selection was performed through a combination of univariate filtering and model-based importance ranking. This pipeline, summarized in Figure 1, ensured that only robust and non-redundant features were retained for model development, thereby improving stability and reproducibility. After segmentation, basic statistical, histogram-based, and textural radiomic features were extracted from each image. The obtained radiomic features were processed independently of the clinical dataset.

Patients and features with a high proportion of missing (null) data were excluded from the study. Four different datasets were created for the final analysis: Clinical, T2W, T1W, and DWI. The number of patients and features in each dataset is presented in Table 2. We acknowledge that sample sizes varied across datasets (161 in CLI and CLI + T2W, 116 in CLI + T1W, 68 in CLI + DWI) due to missing MRI sequences. Analyses using smaller datasets were considered exploratory and interpreted with caution. The CatBoost_CLI dataset, created using clinical data, has the highest sample count, while the addition of radiomics features results in a significant increase in the number of features. This table is essential for evaluating the impact of data diversity and size on model performance comparisons.

### 2.5. Data Cleaning and Preprocessing

Patients and features with a high proportion of missing (null) data were excluded from the study. Features with a missing data rate exceeding 20% and patients with more than 10% missing data were excluded from the analysis [30]. Categorical variables were converted to a numerical format using label encoding before model training. Continuous variables were normalized using the standard scaling method (StandardScaler). These steps were applied to ensure that the model was not affected by variables of different scales and to enable the algorithm to learn more efficiently and stably.

### 2.6. Defining Survival Outcomes

Four different survival outcomes were predicted in the study: Overall survival was defined as the time from biopsy date to the last follow-up or death date. Distant metastasis-free survival (DMFS) was defined as the time from biopsy date to the date of distant metastasis, or if no metastasis was detected, to the last follow-up or death date. Local-regional recurrence-free survival (LRRFS) is defined as the time from the biopsy date to the date of local-regional recurrence, or to the last follow-up or death if no local-regional recurrence is observed. Disease-free survival (DFS) is defined as the time from the biopsy date to the date of local-regional or distant recurrence, whichever occurs first, or to the last follow-up or death if no recurrence is detected. For each survival type, the relevant event column was designated as “Target,” and modelling was performed using this target variable as the basis.

### 2.7. Machine Learning Modelling Process

During the modelling phase, machine learning models were trained using the CatBoost algorithm for each dataset and each survival type. CatBoost is a gradient boosting algorithm that can work directly with categorical variables, is fast, and provides high accuracy [31]. During model training, the dataset was split into 80% training and 20% testing sets. Model hyperparameters were optimized using 5-fold stratified cross-validation [32], which was applied only for parameter tuning and model selection. Final performance was then assessed on the independent 20% hold-out test set. While this approach reduces bias from a single partition, we acknowledge that the absence of repeated hold-out or nested cross-validation and the lack of external validation may increase the risk of overfitting. To address this, model performance was interpreted cautiously, and limitations were explicitly discussed. Model performance was evaluated using metrics such as test accuracy, precision, recall, and F1-score. Additionally, models were further evaluated using ROC curves and Bland–Altman analyses.

Figure 1 summarizes the overall workflow of the machine learning based model developed for predicting survival in LACC. The diagram shows the first step, which is the secure collection of patient data and its storage in a database. Next, data preprocessing steps, such as missing data cleaning and scaling, are performed to prepare the data for analysis. In the next stage, meaningful variables that enhance the model’s predictive power are identified through the selection and extraction of clinical and radiomic features. In the model selection and training phase, advanced machine learning algorithms, such as CatBoost, are employed to develop the most effective model. In the final step, the trained model is used to predict patient survival (life/death) with high accuracy.

This flowchart presents the process from data collection to clinical decision support in a simple, understandable, and systematic manner. Specifically, the integration of radiomics features with clinical data enhances the predictive power of the model and significantly contributes to personalized medicine. Furthermore, the quality control and data processing procedures applied at each step support the model’s generalizability and clinical reliability.

### 2.8. Feature Importances

Feature importance in machine learning models refers to the identification of variables that contribute most to the model’s prediction performance. Feature importance analysis is crucial for understanding the model’s decision-making mechanism, highlighting clinically meaningful variables, and improving the model’s interpretability. Additionally, eliminating unnecessary or low-impact variables helps make the model more straightforward and more generalizable. In clinical applications such as survival prediction, knowing which variables contribute most to predictive power supports both the model’s reliability and clinical decision-making processes.

### 2.9. Software and Reproducibility

All analyses were conducted using open-source Python libraries in a Jupyter Notebook (Conda 24.9.2, Python 3.12.7) environment. The code and analysis pipeline have been archived for reproducibility and transparency.

### 2.10. Statistical Analysis

To compare model performance, metrics such as the area under the ROC curve (AUC—Area Under the Curve), precision, recall, and F1-score were utilized. ROC curves were used to evaluate the discriminative ability of the models. Bland–Altman analysis was applied to evaluate the agreement between model predictions and actual values and to assess whether any systematic bias existed. All analyses were performed in Python (version 3.9) using the scikit-learn (version 1.5.1), pandas (version 2.2.3), NumPy (version 1.24.4), Matplotlib (version 3.7.2), Seaborn (version 0.12.2), and CatBoost (version 1.2.2) libraries. Additionally, IBM SPSS Statistics (version 29; IBM Corp., Armonk, NY, USA) was used for some statistical analyses. Kaplan–Meier analyses were used to assess survival outcomes. This study was approved by the Clinical Research Ethics Committee of Istanbul Faculty of Medicine, Istanbul University (2024–1302). All patient data were anonymized and processed in accordance with privacy principles.

## 3. Results

The median follow-up period was 95 months (range, 18–172 months), with a median age of 52 years (range, 29–84 years). 91.9% of patients were diagnosed with squamous cell carcinoma. The lymph node positivity rate was 46.6% (n = 75), with 44.7% classified as stage I–II according to the FIGO 2018 classification and 55.3% as stage III–IV. Concurrent ChT was administered to 98.8% of the patients, and all patients received EBRT and CT-guided intracavitary 3D-IGABT. The median EBRT dose was 50 Gy (range, 45–52.5), the median number of fractions was 25 (range, 23–30), and the median BT dose was 24 Gy (range, 10–35). The median HR-CTV D90 EQD2(α/β = 10 Gy) dose was 81.7 Gy (range, 62.5–113.6) (Table 1). Disease-free survival is median 94 months (range, 9–168 months), with 5-year and 10-year survival rates of 87.1% (95% CI: 80.64–91.46%) and 84.6% (95% CI: 77.56–89.49%), respectively. Distant metastasis-free survival is median 94 (range, 11–172) months, with 5- and 10-year survival rates of 90.9% (95% CI: 85.08–94.51%) and 89.3% (95% CI: 83.05–93.32%), respectively. Local regional recurrence-free survival is median 94 (range, 9–168) months, with 5-year and 10-year survival rates of 91.4% (95% CI: 85.52–94.89%) and 88.7% (95% CI: 82.16–92.99%), respectively. Overall survival is median 95 (range, 18–172) months, with 5- and 10-year survival rates of 88.9% (95% CI: 82.73–92.95%) and 76.4% (95% CI: 67.53–82.91%), respectively.

Table 3A lists the top 10 most essential features in the model created using only clinical variables. Variables such as “tumor diameter,” “HR-CTV D90 EQD2_10Gy_,” and “HR-CTV” emerge as the most decisive factors in the model’s survival prediction. These findings confirm that classical clinical variables remain essential prognostic factors, while the integration of radiomics—particularly morphological and textural descriptors from T2W imaging—adds complementary information reflecting tumor heterogeneity. Clinical parameters, such as the presence of comorbidities, the number of concurrent chemotherapies, and age, also significantly contribute to the predictive power of the model, particularly in cases with high scores. These results demonstrate that classical clinical variables remain the primary determinants in survival prediction and play an essential role in the decision-making mechanism of the model.

In Table 3B, the top 10 features with the highest importance in the model, which include T1W sequence radiomics features, are listed. At the top of the list are morphological and intensity-based radiomics parameters.

In particular, morphological measures such as “MORPHOLOGICAL_RadiusSphereNorm-MaxIntensityCoor” and “MORPHOLOGICAL_Maximum3DDiameter,” along with tissue and intensity analysis-based features like “GLCM_AngularSecondMoment” and “INTENSITY-BASED_IntensityVariance,” play a decisive role in the model’s survival prediction.

Among clinical variables, only “number of concurrent ChT cycles” made it into the top 10. This table demonstrates that the inclusion of radiomics data enhances the predictive power of image-based features in survival prediction, thereby improving the model’s predictive ability. Notably, morphological and tissue features contribute significantly to survival prediction when used in conjunction with clinical data.

Table 3C lists the top 10 features with the highest importance in the model created by adding radiomics features to clinical variables using the T2W sequence. At the top of the list are intensity- and tissue-based radiomics parameters such as “INTENSITY-BASED_IntensityBasedQuartileCoefficient” and “NGTDM_Contrast.” Additionally, morphological and tissue analysis features such as “MORPHOLOGICAL_RadiusRoiNorm-MaxIntensityCoor” and “GLRLM_LongRunsEmphasis” also play a significant role in the model’s survival prediction. Among clinical variables, only “age” made it into the top 10. This table demonstrates that image-based features significantly outperform clinical features in survival prediction when T2W sequence radiomics features are added to the model. In particular, intensity, texture, and morphological parameters enhance the model’s predictive power when used in conjunction with clinical data.

Table 3D lists the top 10 most essential features in the model created by adding DWI sequence radiomic features to clinical variables. At the top of the list is a clinical variable, such as “number of concurrent ChT cycles,” followed by morphological and tissue-based radiomics parameters, including “MORPHOLOGICAL_Maximum3DDiameter” and “GLRLM_LongRunHighGreyLevelEmphasis.” Additionally, various radiomics features, such as “GLSZM_GreyLevelVariance,” “MORPHOLOGICAL_Compacity,” and “GLCM_Autocorrelation,” also play a significant role in the model’s survival prediction. This table demonstrates that both clinical and image-based features are helpful in survival prediction when DWI radiomics features are incorporated into the model. In particular, the morphological and tissue analysis parameters enhance the predictive power of the model when integrated with clinical data.

Table 3 demonstrates that incorporating radiomics data with clinical data into survival prediction can significantly enhance the predictive power of the model, and that image-based parameters can be a valuable tool in clinical decision support systems.

Table 4A presents the performance metrics of models trained using only clinical data. In this setting, the highest performance was observed for LRRFS (test accuracy 84.38%, F1-score 80.08) and OS (accuracy 78.12%, F1-score 71.27). DFS (accuracy 71.88%, F1-score 72.89) and DMFS (accuracy 71.88%, F1-score 73.18) showed moderate results. These findings indicate that clinical data alone provide a solid but limited foundation for survival prediction.

Table 4B presents the model results obtained by adding T1W sequence radiomics features to clinical data. Compared to clinical data alone, the addition of T1W features yielded a clear improvement in DFS (test accuracy 86.96%, F1-score 80.89), LRRFS and DMFS (accuracy 91.30%, F1-score 87.15). However, the OS performance remained relatively limited (accuracy 69.57%, F1-score 64.21). These findings show that T1W radiomics can contribute to specific survival endpoints, particularly DFS, LRRFS and DMFS.

Table 4C presents the model performance obtained by adding T2W sequence radiomics features to clinical data. The best results in this group were obtained for DMFS (test accuracy 92.31%, F1-score 88.62). Consistently high performance was also achieved for DFS (accuracy 84.62%, F1-score 77.56) and LRRFS (accuracy 84.62%, F1-score 77.56). However, the OS performance remained relatively limited (accuracy 76.92%, F1-score 73.58). These results demonstrate that T2W sequence radiomics features provide a strong contribution, especially for distant metastasis prediction.

Table 4D presents the model results obtained by adding DWI radiomic features to clinical data. Compared to T1W and T2W models, the DWI-based model showed slightly lower performance in DMFS (accuracy 87.50%, F1-score 81.67) and OS (accuracy 75.00%, F1-score 66.96). Nevertheless, high performance was maintained for DFS (accuracy 84.38%, F1-score 77.22) and LRRFS (accuracy 82.41%, F1-score 77.41). This suggests that DWI radiomics can contribute to DFS and LRRFS prediction, but adds less prognostic value for DMFS and OS.

The best model results obtained using the CatBoost algorithm are summarized in Table 4. The highest accuracy and F1-score values were achieved in models that combined clinical and radiomic data, particularly with T1W and T2W sequences. In this analysis, the CatBoost_CLI + T1_DMFS (accuracy 91.30%, F1-score 87.15) and CatBoost_CLI + T2_DMFS (accuracy 92.31%, F1-score 88.62) models stood out as the strongest predictors.

**Figure 2:**
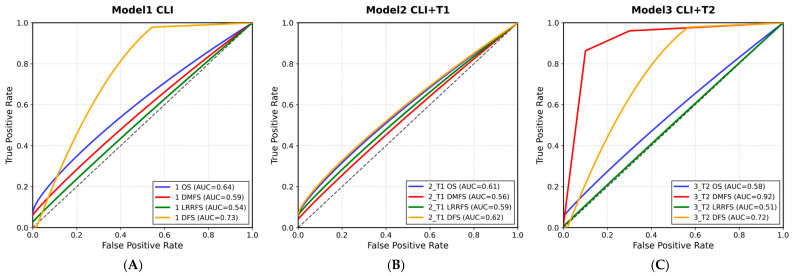
(**A**): ROC curve for Model 1, (**B**): ROC curve for Model 2, (**C**): ROC curve for Model 3.

**Figure 3:**
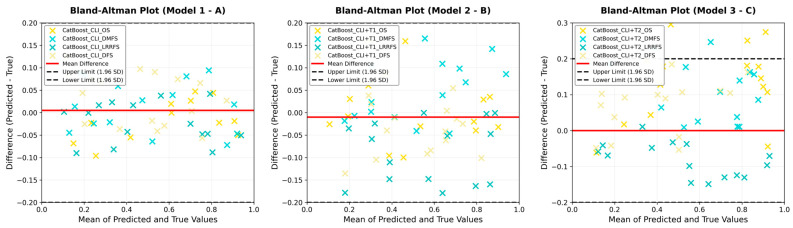
(**A**): Bland–Altman plot for Model 1, (**B**): Bland–Altman plot for Model 2, (**C**): Bland–Altman plot for Model 3.

The ROC curves of the model performances are presented in Figure 2, and the Bland–Altman analyses are presented in Figure 3. While AUC values for DFS, DMFS, and LRRFS endpoints in the clinical-only model were moderate (DFS = 0.73, DMFS = 0.59, LRRFS = 0.54), the discriminative ability for OS was limited (AUC = 0.64). The integration of radiomics features from T1W (OS AUC = 0.61) and T2W (OS AUC = 0.58) sequences did not markedly improve the OS performance. In contrast, the T2W-based model demonstrated a substantial gain for DMFS (AUC = 0.92). These results suggest that radiomics contributes more specifically to distant metastasis prediction, whereas improvements for OS, DFS, and LRRFS endpoints were modest. The ROC curves show that the models have high discriminative power (high AUC values). The Bland–Altman analyses indicate that there is no systematic deviation in the model predictions and that the predictions are reliable [33].

The ROC curves of the model performances are presented in Figure 2, and the Bland–Altman analyses are presented in Figure 3. The ROC curves show that the models have high discriminative power (high AUC values). The Bland–Altman analyses indicate that there is no systematic deviation in the model predictions and that the predictions are reliable [33].

Figure 2A shows the ROC (Receiver Operating Characteristic) curve obtained for Model 1 on the test data. The ROC curve visually displays the sensitivity and specificity performance of the model at different threshold values. The AUC value is a critical metric summarizing the model’s classification performance. In Figure 2A, the ROC curve’s proximity to the ideal corner in the upper left corner indicates that Model 1 has high discriminative power and performs well in survival prediction. In particular, the high AUC value indicates that the model successfully distinguishes between positive and negative classes.

Figure 2B shows the ROC curve obtained for Model 2 on the test data. The ROC curve illustrates the balance between sensitivity and specificity at different threshold values. The curve’s proximity to the upper left corner indicates that Model 2 has high accuracy and discriminative ability. The high AUC value indicates that the model can successfully distinguish between positive and negative classes, providing reliable results in survival prediction. The ROC curve of Model 2 supports that clinical and radiomic data strengthen the model and have excellent prediction performance.

Figure 2C shows the ROC curve obtained for Model 3 on the test data. The ROC curve compares the sensitivity and specificity performance of the model at different threshold values. The curve’s proximity to the upper left corner indicates that Model 3 has high discriminative power and demonstrates successful performance in survival prediction. A high AUC value indicates that the model can effectively distinguish between positive and negative classes and can be used reliably in clinical applications. The ROC curve of Model 3 visually supports the model’s overall accuracy and reliability.

The primary reason for the ROC curve and AUC values of Models 4 being “nan” (not a number) is the very low number of samples used in these models. The ROC curve and AUC value require a sufficient number of examples to evaluate the model’s ability to distinguish between positive and negative classes. However, when the sample size is small, it is not possible to measure the performance of the model in a statistically significant and reliable way. This situation becomes more pronounced in cases of imbalanced datasets or when the class distribution is very low. As a result, the small sample size in Models 4 and 5 prevented the calculation of the ROC curve and AUC score, resulting in the reported results being marked as “nan.” Therefore, larger and more balanced datasets are required for the performance evaluation of these models.

The ROC curves obtained in the study were used to evaluate the predictive performance of the survival models. The ROC curve visually demonstrates the model’s ability to distinguish between positive and negative classes at different threshold values. In the figure, the ROC curves of each model and their corresponding AUC values are presented. AUC values above 0.90 indicate that the models have high discriminative power and provide reliable results in survival prediction.

In particular, the fact that the ROC curves are located in the upper region of models that use clinical and radiomic data together indicates that this data combination enhances prognostic accuracy. These results indicate that clinical data alone (Model 1) provide partial prognostic value (OS AUC = 0.64, DFS AUC = 0.73), while the addition of T1 radiomic features (Model 2, AUC range 0.56–0.62) does not yield a substantial improvement. In contrast, the integration of T2 radiomic features (Model 3) shows a marked contribution, particularly in predicting distant metastasis (DMFS, AUC = 0.92), suggesting that the combination of clinical and T2 radiomic data substantially enhances prognostic accuracy.

Figure 3A shows the Bland–Altman plot created to evaluate the agreement between the predicted values of Model 1 and the actual values. The Bland–Altman analysis visually presents the average difference between two measurement methods and the distribution of these differences. In the figure, it can be seen that the majority of the points are clustered around the mean difference and within the limits of agreement. This indicates that Model 1’s predictions are highly consistent with the actual values and do not show systematic bias. Additionally, the scarcity of outliers and the homogeneous distribution of points support the reliability and consistency of the model. The Bland–Altman plot demonstrates that Model 1 provides statistically significant and clinically acceptable accuracy in survival estimates.

Figure 3B shows the Bland–Altman plot created to evaluate the agreement between the values predicted by Model 2 and the actual values. The plot indicates that the differences between the expected and actual values are primarily centered around the mean difference and within acceptable limits. This suggests that the predictions of Model 2 are generally consistent with the exact values and do not show systematic deviation. The fact that most of the points remain within the limits supports the high predictive power and reliability of the model. Additionally, the scarcity of outliers and the homogeneous distribution of differences indicate that Model 2 provides stable and reliable results in survival predictions.

Figure 3C shows the Bland–Altman plot created to evaluate the agreement between the values predicted by Model 3 and the actual values. The graph shows that the majority of differences between predicted and actual values are centered around the mean difference and within acceptable limits. This indicates that Model 3’s predictions are generally consistent with exact values and that the model does not exhibit systematic bias. Additionally, the homogeneous distribution of points within the limits supports the high predictive power and reliability of the model. The Bland–Altman analysis demonstrates that Model 3 provides statistically significant and clinically acceptable accuracy in survival predictions.

Bland–Altman plots could not be created for Models 4 and 5 because the sample size in these models was insufficient for statistical analysis. Bland–Altman analysis requires a certain number of data points to meaningfully assess the distribution of differences between two measurements and their confidence intervals. When the sample size is very low, both the mean difference and the limits of agreement cannot be reliably calculated, and the graph loses its meaning. Therefore, larger and more balanced datasets are needed to evaluate the prediction performance of Models 4 and 5.

The Bland–Altman plot obtained in this study was used to assess the agreement between the survival predictions of machine learning models and actual observations. In the graph, the horizontal axis represents the average of the model predictions and the exact values, while the vertical axis shows the differences between them. The fact that the majority of the points are clustered around the average difference and within the ±1.96 standard deviation limits (limits of agreement) indicates that the model predictions are highly consistent with the actual values. Additionally, the average difference being close to zero indicates that there is no systematic bias in the models and that the predictions are balanced. These results support the reliability and consistency of the machine learning models developed for survival predictions.

## 4. Discussion

In our study, when clinical data and radiomics data obtained from MRI were analyzed in patients with LACC treated with CRT and CT-based adaptive brachytherapy, the highest accuracy and F1-score values were obtained in models combining clinical and radiomics data, particularly from T1W and T2W sequences. In this analysis, the CatBoost_CLI + T1_DMFS (accuracy 91.30%, F1-score 87.15) and Cat-Boost_CLI + T2_DMFS (accuracy 92.31%, F1-score 88.62) models stood out as the strongest predictors.

ROC analysis shows that clinical data alone (Model 1) provides partial prognostic value (OS AUC = 0.64, DFS AUC = 0.73), while the addition of T1 radiomics features (Model 2, AUC range 0.56–0.62) does not provide a significant improvement. In contrast, the integration of T2 radiomics features (Model 3) shows a significant contribution, particularly in the prediction of distant metastasis (DMFS, AUC = 0.92), suggesting that combining clinical and T2 radiomics data significantly improves prognostic accuracy.

Since 1999, concurrent CRT and BT have been considered the standard of care for the curative treatment of locally advanced cervical cancer. With technological advancements, both EBRT and BT have transitioned from 2D to 3D treatment methods. In fact, MRI-based IGABT is considered the gold standard for BT. Cervical cancer is more common in countries with low socioeconomic status and limited resources. Brachytherapy is ineligible for patients due to the lack of treatment equipment, accessibility, or patient or disease characteristics. According to Kumar et al., over the past few decades, approximately 50% of patients in the United States have been treated without the use of BT [34]. Ineligibility for brachytherapy can occur for a variety of reasons, including anatomical, medical, technical, and patient-related factors. Retrospective series, phase I/II studies, reviews, and meta-analyses emphasize that stereotactic body radiotherapy (SBRT) may be an alternative treatment for patients not suitable for brachytherapy [35,36,37,38,39,40,41].

Prognosis prediction in LACC has been investigated in various nomograms, scoring, and artificial intelligence studies to improve treatment outcomes and assist in treatment selection. Current nomograms include age, race, marital status, grade, histological type, T stage, N stage, M stage, RT, tumor size, FIGO stage, maximum length of primary tumor, chemotherapy, squamous cell carcinoma (SCC-Ag), neutrophil-to-lymphocyte ratio, city of residence, and household income [42,43,44,45,46]. Still, the nomogram parameters consist of parameters other than tumor, imaging, and treatment characteristics, or a small number of parameters. Sturdza et al. constructed the first nomogram to predict OS in patients with LACC treated with IGABT, including FIGO Stage 2009, lymph node involvement, concurrent chemotherapy, HR-CTV volume in the first fraction BT, corpus involvement, total treatment duration, age at diagnosis, and lymph node status. They created a web-based nomogram [12]. In their research using single-center data, Ibis et al. demonstrated that the nomogram developed by Sturdza et al. underestimates the 60-month overall survival rate [47]. Lindegaard et al. used pretreatment MRI and gynecologic examination data from 400 LACC patients treated with CRT and IGABT to determine the involvement of the cervix, left parametrium, right parametrium, vagina, bladder, ureter, rectum, and uterine corpus. These data were scored on an ordinal scale of 0 to 3 points and were used to obtain the total tumor score. As the tumor score increases, both at the time of diagnosis and at the time of brachytherapy, overall survival and local control rates decrease [13,48].

Another essential tool in estimating the prognosis of cervical cancer is radiomics analysis. T2-weighted sequences are crucial in the MRI evaluation of cervical cancer. Clinical target volume definition guidelines for IMRT in the definitive treatment of cervical cancer also describe the characteristics of the tumor and surrounding tissue invasion using the T2-weighted sequence MRI [49]. Therefore, prognostic information obtained from the T2W sequence MRI at the time of diagnosis is essential and valuable. In our study, we achieved the most successful results across all outcomes in the model where we added radiomic data obtained from T2W sequence MRI images to clinical data.

In radiomics analyses, three-dimensional MRI features obtained from the tumor and surrounding tissue can predict DFS and OS with high accuracy [16]. Xin et al. retrospectively analyzed 700 patients with IB2-IVA cervical cancer who underwent CRT and examined radiomic features in T2-weighted MRI sequences. They reported that machine learning-based radiomic models can assist in predicting DFS and OS after concurrent CRT in patients with LACC, and that the results could provide a reliable basis for treatment decision-making in cervical cancer patients [16]. In our study, the CLI + T2W model (Model 3) achieved the highest predictive performance across all outcomes (DFS, DMFS, LRRFS, OS), with DMFS reaching 92.31% accuracy and 88.62% F1-score. This highlights the dominant contribution of T2W radiomic features when integrated with clinical variables.

Meng et al. conducted a prospective study to investigate the effects of tissue properties derived from T2W and apparent diffusion coefficient (ADC) maps on recurrence prediction in patients with LACC treated with CRT. They performed pelvic MRI on 34 patients before, during, and after CRT. They extracted radiomic features using software on T2W and ADC maps and evaluated their performance in predicting recurrence. They reported that data obtained from ADC tissue parameters showed the best performance in predicting recurrence, while the combination of T2W tissue parameters may add little value to prognosis [50]. In our study, DWI radiomics programs combined with clinical data yielded less successful results, and we acknowledge that the sample size was small for generalizability. Because we aimed to present real-world data in this retrospective study, we considered excluding DWI sequence data.

Sittiwong et al. used T2W and DWI sequences in pre-treatment and pre-BT MRI of 100 patients treated with CRT and 3D-IGABT to extract radiomic characteristics of primary tumors and lymph nodes. They reported that radiomic features added value to clinical factors in predicting outcomes after CRT, with the highest model performance achieved by combining radiomic data from MRI scans before EBRT and BT with clinical data [51]. Zhang et al. retrospectively analyzed 198 patients with LACC who underwent CRT. Radiomic features were obtained from T2W imaging and visible diffusion coefficient maps on MRI performed one month after completion of CRT and evaluated in conjunction with clinical data. Among clinical variables, tumor grade and FIGO stage were identified as independent risk factors. Data obtained from radiomic features were reported to predict treatment response better than clinical data, although this difference was not statistically significant [52]. In our study, when radiomic and clinical data obtained from MRI images taken before EBRT were analyzed during a median follow-up period of 95 months (18–172), it was observed that the addition of radiomic features, particularly T1W and T2W-, significantly improved the predictive power for DFS, DMFS, LRRFS, and OS. Although the number of patients in our study was relatively limited, we believe that it is valuable due to the median follow-up period of 7.92 years.

In recent years, the number of studies conducted in the clinical field using machine learning (ML) and deep learning (DL) models has increased exponentially due to their potential to assist clinical decision-making processes such as early warning, treatment personalization, and improvement of clinical trial outcomes [8,50,51,53]. Vazquez et al. reported in their review that 54.9% of clinical applications in the reviewed literature were related to diagnosis, 22.9% to prognosis, and 22.2% to treatment. They emphasized that most studies focused on predicting cancer progression using CT and MRI for prognosis. Vazquez et al. reported that China has the highest number of studies, with a total of 85 studies (40 diagnostic, 21 prognostic, and 24 treatment-focused), followed by the United States with 30 studies (16 diagnostic, four prognostic, and 10 treatment-focused), India conducted 12 studies, and the Republic of Korea conducted nine studies. They reported that countries with fewer studies, including Türkiye, provided recommendations for cervical cancer diagnosis [53]. In light of these findings, our study is noteworthy because it evaluates the prognosis of LACC, which has been studied to a lesser extent, has a long follow-up period, provides detailed patient and treatment characteristics, and offers insights into prognosis prediction in a sample with few studies on this topic.

In our study, preprocessing steps such as incomplete data cleaning, feature engineering, and scaling, which were applied during the model development process, increased the generalizability and reliability of the models. The fact that all analyses were performed using open-source Python libraries supports the transparency and reproducibility of the study. Furthermore, when examining feature importance, it was observed that clinical variables, such as survival times and event statuses, as well as radiomics-based morphological and intensity features, contributed significantly to the model’s performance. These findings strengthen the usability of radiomics data in clinical decision support systems.

However, the study has some limitations. The relatively small and inconsistent sample sizes in certain subsets (DWI) limited our ability to perform some statistical analyses such as ROC/AUC evaluation, reducing the strength of these exploratory results. The very high-performance metrics observed in some models may also reflect potential overfitting, given the single-center retrospective design, the relatively limited number of patients, and the reliance on a single 20% hold-out test set without external validation. Although five-fold stratified cross-validation was employed during hyperparameter tuning to reduce partition bias, repeated or nested cross-validation and external cohort validation would provide more substantial evidence for model generalizability. Future studies with larger, multi-institutional datasets are therefore warranted to validate and extend our findings. In particular, the reliability of model performance and statistical analyses decreases in datasets with low sample sizes. Therefore, validating the obtained results with multicenter, larger, and prospective datasets is crucial for integrating radiomics-based machine learning models into clinical practice.

Machine learning-based clinical and radiomics models offer higher accuracy and risk factor identification capabilities compared to traditional methods in predicting survival in LACC. The integration of clinical and imaging data can provide significant contributions to personalized treatment planning and patient follow-up. However, further research is needed on model transparency and its widespread clinical application. In addition, the clinical applicability of our model deserves emphasis. Prognostic estimates generated before treatment could assist clinicians in tailoring strategies for high-risk patients, such as considering neoadjuvant chemotherapy, immunotherapy, or radiotherapy dose escalation. The model may also support patient monitoring by informing risk-adapted follow-up schedules. From an implementation perspective, integration through a web-based AI service layer connected to PACS or oncology information systems would allow radiomics features to be automatically extracted from MRI scans and predictions delivered directly to the clinician’s workstation. This workflow could facilitate seamless use in daily practice and contribute to more personalized and adaptive treatment planning. Furthermore, it is necessary and proper that this prognostic information can be obtained using the T2W sequence, the most basic and essential MRI sequence for the radiological evaluation of cervical cancer, which radiation oncologists can most easily evaluate.

## 5. Conclusions

In this study, CatBoost-based machine learning models were developed to predict survival in patients with LACC using clinical and radiomic features. The integration of radiomics, particularly from T2W sequences, provided notable improvements in predictive accuracy compared to clinical data alone. However, the very high-performance metrics observed in certain models must be interpreted cautiously, as they may partly reflect overfitting due to the single-center retrospective design, relatively small and inconsistent sample sizes in subsets, and reliance on a single hold-out test set. The inability to perform key statistical analyses in smaller datasets further underscores the importance of larger, balanced cohorts. Therefore, while our findings are promising, external multicenter validation and more robust statistical approaches are required before clinical implementation.

## Figures and Tables

**Table 1:** Baseline patient characteristics and treatment characteristics (*n* = 161).

Patient Characteristics		Treatment Characteristics	
Characteristics	Numbers (%) Median (Min.–Max.)	Characteristics	Numbers (%) Median (Min.–Max.)
Age (years)	*n* = 161 52 (29–84)	EBRT technique 3D-CRT IMRT/VMAT	*n* = 161 93 (57.8%) 68 (42.2%)
KPS score	*n* = 161 100 (70–100)	Total EBRT doses	*n* = 161 50 (45–52.5)
Pretreatment haemoglobin (g/dL)	*n* = 136 12.05 (7.2–15.2)	Total EBRT fractions	*n* = 161 25 (23–30)
Neutrophil/lymphocyte ratio	*n* = 130 3.06 (1.21–40.20)	Total brachytherapy dose	*n* = 161 24 (10–35)
Menopause status Postmenopause Premenopause Perimenopause	*n* = 161 89 (55.3%) 64 (39.8%) 8 (5%)	Brachytherapy fractions	*n* = 161 4 (2–6)
Pathology Squamous cell carcinoma Adenocarcinoma Serous papillary carcinoma	*n* = 161 148 (91.9%) 12 (7.4%) 1 (0.6%)	HR-CTV cc	*n* = 154 29.95 (4.33–74.76)
Tumor diameter	*n* = 160 4.7 2–12	HR-CTV group ≤30 cc >30 cc Unknown	*n* = 161 77 (47.8%) 77 (47.8%) 7 (4.3%)
Tumor diameter ≤2 cm 2.1–4 cm >4 cm Unknown	*n* = 161 2 (1.2%) 48 (29.8%) 110 (68.3%) 1 (0.6%)	HR-CTV D98 EQD2_α/β=10Gy_	*n* = 153 73.9 59.50–97.80
18F-FDG-PET/CT SUVmax	*n* = 148 16 (6–57)	HR-CTV D90 EQD2_α/β=10Gy_	*n* = 161 81.7 (62.5–113.6)
Involved lymph node Yes No	*n* = 161 75 (46.6%) 86 (53.4%)	Right A point D90 EQD2_α/β=10Gy_	*n* = 153 66.3 (53.5–89.70)
Involved lymph node region Pelvic region Paraaortic region Pelvic + paraaortic region No	*n* = 161 64 (39.8%) 2 (1.2%) 9 (5.6%) 86 (53.45)	Left A point D90 EQD2_α/β=10Gy_	*n* = 153 67 (53.5–89.8)
EBRT response Complete response Residue Unknown	*n* = 161 71 (44%) 86 (53.4%) 4 (2.5%)	Total treatment time (days)	*n* = 161 82 (52–212)
FIGO2018 Staging IB2 IB3 IIA1 IIB IIIA IIIB IIIC1 IIIC2 IVA	*n* = 161 5 (3.1%) 1 (0.6%) 1 (0.6%) 65 (40.4%) 2 (1.2%) 11 (6.8%) 61 (37.9%) 11 (6.8%) 4 (2.5%)	Total treatment time group ≤80 days >80 days	*n* = 161 76 (47.2%) 85 (52.8%)
		Concurrent chemotherapy drugs Cisplatin Carboplatin Low-dose paclitaxel-carboplatin No	*n* = 161 154 (95.7%) 1 (0.6%) 4 (2.5%) 2 (1.2%)
FIGO2018 Staging Group Stage I–II Stage III–IV	*n* = 161 72 (44.7%) 89 (55.3%)	Concurrent chemotherapy cycles	*n* = 161 4 (0–7)

KPS: Karnofsky performance status; 18F-FDG-PET/CT: 18F-fluorodeoxyglucose positron emission tomography/computed tomography–maximum standard uptake value; EBRT: External beam radiation therapy; FIGO: The International Federation of Gynaecology and Obstetrics; 3D-CRT: 3-dimensional conformal radiation therapy; IMRT: Intensity modulated radiation therapy; VMAT: Volumetric modulated arc therapy; HR-CTV: High risk-clinical target volume; EQD2: 2 Gy equivalent dose; Gy: Gray.

**Table 2:** Characteristics of the Datasets.

Dataset(s)	Sample Size	Feature Size
CatBoost_CLI	161	55
CatBoost_CLI + T1W	116	214
CatBoost_CLI + T2W	161	214
CatBoost_CLI + DWI	68	214

**Table 3:** Feature Importance Scores in Different Models and Data Combinations.

**(A) Model with Clinical Features (CatBoost_CLI)**		
**No**	**Feature(s)**	**Score**
1	Tumor_diameter_cm	0.067731
2	HR-CTV D90 EQD2_10Gy_	0.058711
3	HR-CTV Volume	0.043819
4	Comorbid condition	0.043310
5	Number of concurrent chemotherapy cycles	0.042640
6	R–A point EQD2_10Gy_	0.041074
7	HR-CTV D98 EQD2_10Gy_	0.040643
8	Age	0.039858
9	L–A_point EQD2_10Gy_	0.037941
10	Pre-treatment 18F-FDG-PET/CT-SUVmax	0.036313
**(B) Clinical + T1W Radiomics Features (CatBoost_CLI + T1)**		
**No**	**Feature(s)**	**Score**
1	MORPHOLOGICAL_RadiusSphereNorm-MaxIntensityCoo	0.036061
2	MORPHOLOGICAL_Maximum3DDiameter(IBSI:L0JK) [mm]	0.028205
3	GLCM_AngularSecondMoment(IBSI:8ZQL)	0.017962
4	INTENSITY-BASED_IntensityVariance(IBSI:ECT3)	0.017237
5	GLRLM_GreyLevelVariance(IBSI:8CE5)	0.015982
6	GLCM_NormalisedInverseDifferenceMoment(IBSI:1QCO)	0.015428
7	GLSZM_SmallZoneHighGreyLevelEmphasis(IBSI:HW1V)	0.014849
8	Number of concurrent chemotherapy cycles	0.014651
9	MORPHOLOGICAL_RadiusSphereNorm-MaxIntensityCoo	0.013918
10	GLSZM_ZoneSizeNonUniformity(IBSI:4JP3)	0.013632
**(C) Clinical + T2W Radiomic Features (CatBoost_CLI + T2)**		
**No**	**Feature(s)**	**Score**
1	INTENSITY-BASED_IntensityBasedQuartileCoeffici	0.029127
2	NGTDM_Contrast(IBSI:65HE)	0.023912
3	MORPHOLOGICAL_RadiusRoiNorm-MaxIntensityCoor	0.018926
4	GLRLM_LongRunsEmphasis(IBSI:W4KF)	0.017441
5	GLCM_Contrast(IBSI:ACUI)	0.016675
6	GLRLM_RunEntropy(IBSI:HJ90)	0.015700
7	INTENSITY-BASED_IntensityBasedEnergy(IBSI:N8CA)	0.015531
8	Age	0.015137
9	INTENSITY-HISTOGRAM_IntensityHistogramSkewness	0.013715
10	INTENSITY-BASED-RIM_RIM-IntensityMean(IBSI:No)	0.013389
**(D) Clinical + DWI Radiomics Features (CatBoost_CLI + DWI)**		
**No**	**Feature(s)**	**Score**
1	Number of concurrent chemotherapy cycles	0.026246
2	MORPHOLOGICAL_Maximum3DDiameter(IBSI:L0JK) [mm]	0.024132
3	GLRLM_LongRunHighGreyLevelEmphasis(IBSI:3KUM)	0.022260
4	GLSZM_GreyLevelVariance(IBSI:BYLV)	0.021811
5	MORPHOLOGICAL_Compacity(IBSI:No)	0.020744
6	GLCM_Autocorrelation(IBSI:QWB0)	0.019438
7	GLSZM_ZoneSizeNonUniformity(IBSI:4JP3)	0.019039
8	INTENSITY-BASED-RIM_RIM-CountingVoxels(IBSI:No)	0.018038
9	MORPHOLOGICAL_MaxIntensityCoor-PerimeterCoor-2	0.017279
10	INTENSITY-BASED_IntensityKurtosis(IBSI:IPH6)	0.016723

HR-CTV: High risk-clinical target volume; EQD2: 2 Gy equivalent dose; Gy: Grey, 18F-FDG-PET/CT-SUVmax: 18F-fluorodeoxyglucose positron emission tomography/computed tomography–maximum standard uptake value.

**Table 4:** Performance Metrics of Models with Different Clinical and Radiomic Feature Combinations.

**(A) Model Performance Obtained from Clinical Data (Model 1: CatBoost_CLI)**				
**Model(s)**	**Test_Accuracy (%)**	**Precision (%)**	**Recall (%)**	**F1_Score (%)**
CatBoost_CLI_DFS	71.88	74.00	71.88	72.89
CatBoost_CLI_DMFS	71.88	74.54	71.88	73.18
CatBoost_CLI_LRRFS	84.38	76.21	84.38	80.08
CatBoost_CLI_OS	78.12	65.52	78.12	71.27
**(B) Clinical + T1W Radiomic Features with Model Performance (Model 2: CatBoost_CLI + T1W)**				
**Model(s)**	**Test_Accuracy (%)**	**Precision (%)**	**Recall (%)**	**F1_Score (%)**
CatBoost_CLI + T1_DFS	86.96	75.61	86.96	80.89
CatBoost_CLI + T1_DMFS	91.30	83.36	91.30	87.15
CatBoost_CLI + T1_LRRFS	86.96	75.61	86.96	80.89
CatBoost_CLI + T1_OS	69.57	59.63	69.57	64.21
**(C) Clinical + T2W Radiomic Features with Model Performance (Model 3: CatBoost_CLI + T2W)**				
**Model(s)**	**Test_Accuracy (%)**	**Precision (%)**	**Recall (%)**	**F1_Score (%)**
CatBoost_CLI + T2_DFS	84.62	71.60	84.62	77.56
CatBoost_CLI + T2_DMFS	92.31	85.21	92.30	88.62
CatBoost_CLI + T2_LRRFS	84.62	71.60	84.62	77.56
CatBoost_CLI + T2_OS	76.92	70.51	76.92	73.58
**(D) Model Performance with Clinical + DWI Radiomic Features (Model 4: CatBoost_CLI + DWI)**				
**Model(s)**	**Test_Accuracy (%)**	**Precision (%)**	**Recall (%)**	**F1_Score (%)**
CatBoost_CLI + DIFF_DFS	84.38	71.19	84.38	77.22
CatBoost_CLI + DIFF_DMFS	87.50	76.56	87.50	81.67
CatBoost_CLI + DIFF_LRRFS	82.41	73.12	82.25	77.41
CatBoost_CLI + DIFF_OS	75.00	60.48	75.00	66.96

CLI: Clinic; CLI + T1W: Clinic + contrast-enhanced T1-weighted; CLI + T2W: Clinic + T2-weighted; CLI + DWI: Clinic + Diffusion-weighted imaging; LRRFS: Locoregional-free survival; DMFS: Distant metastasis-free survival; DFS: Disease-free survival, OS: Overall survival.

## Data Availability

The raw data supporting the conclusions of this article will be made available by the authors on request.

## Abbreviations

The following abbreviations are used in this manuscript:

18F-FDG-PET/CT	18F-fluorodeoxyglucose positron emission tomography/computed tomography–maximum standard uptake value
3D-CRT	3-dimensional conformal radiation therapy
ADC	Apparent diffusion coefficient
AUC	Area under the curve
BT	Brachytherapy
ChT	Chemotherapy
CLI	Clinic
CLI + DWI	Clinic + Diffusion-weighted imaging
CLI + T1W	Clinic + contrast-enhanced T1-weighted
CLI + T2W	Clinic + T2-weighted
CRT	Chemoradiotherapy
CT	Computed tomography
DFS	Disease-free survival
DMFS	Distant metastasis-free survival
EBRT	External beam radiation therapy
EQD2	2 Gy equivalent dose
FIGO	The International Federation of Gynaecology and Obstetrics
Gd-DTPA	Gadopentetate glucosamine injection
Gy	Gray
HPV	Human papillomavirus
HR-CTV	High-risk clinical target volume
IBSI	Image Biomarker Standardization Initiative
IGABT	Guided adaptive brachytherapy
IMRT	Intensity modulated radiation therapy.
KPS	Karnofsky performance status
LACC	Locally advanced cervical cancer
LRRFS	Local-regional recurrence-free survival
MRI	Magnetic resonance imaging
OS	Overall survival
PACS	Picture Archiving and Communication System
ROC	Receiver Operating Characteristic
RT	Radiotherapy
VMAT	Volumetric modulated arc therapy
